# Retraction: Stimulator of Interferon Genes in Classical Dendritic Cells Controls Mucosal Th17 Responses to Cyclic Dinucleotides for Host Defenses Against Microbial Infections in Gut

**DOI:** 10.3389/fimmu.2019.01468

**Published:** 2019-06-14

**Authors:** Frontiers Editorial Office

The journal and Chief Editors retract the 16 May 2018 article cited above.

Following the publication of the article, concerns regarding the source of the data used in the article were addressed to the journal and its Editorial Board. Following clarification with the authors, it was identified that data generated at a different institute were partially included in their article erroneously. The article is therefore being retracted.

The authors concur with the retraction and sincerely regret any inconvenience this may have caused to the reviewers, editors, and readers of Frontiers in Immunology.

